# Genomic mechanisms of plant growth-promoting bacteria in the production of leguminous crops

**DOI:** 10.3389/fgene.2023.1276003

**Published:** 2023-11-03

**Authors:** Afeez Adesina Adedayo, Olubukola Oluranti Babalola

**Affiliations:** Food Security and Safety Focus Area, Faculty of Natural and Agricultural Sciences, North-West University, Mmabatho, South Africa

**Keywords:** abiotic stress, genome editing, genotyping, PGPR interaction, plant breeding

## Abstract

Legumes are highly nutritious in proteins and are good food for humans and animals because of their nutritional values. Plant growth-promoting bacteria (PGPR) are microbes dwelling in the rhizosphere soil of a plant contributing to the healthy status, growth promotion of crops, and preventing the invasion of diseases. Root exudates produced from the leguminous plants’ roots can lure microbes to migrate to the rhizosphere region in other to carry out their potential activities which reveals the symbiotic association of the leguminous plant and the PGPR (rhizobia). To have a better cognition of the PGPR in the rhizosphere of leguminous plants, genomic analyses would be conducted employing various genomic sequences to observe the microbial community and their functions in the soil. Comparative genomic mechanism of plant growth-promoting rhizobacteria (PGPR) was discussed in this review which reveals the activities including plant growth promotion, phosphate solubilization, production of hormones, and plant growth-promoting genes required for plant development. Progress in genomics to improve the collection of genotyping data was revealed in this review. Furthermore, the review also revealed the significance of plant breeding and other analyses involving transcriptomics in bioeconomy promotion. This technological innovation improves abundant yield and nutritional requirements of the crops in unfavorable environmental conditions.

## Introduction

The reduction in food crop production nowadays results from the inability to tame wild species, the potential for average condition of weather, and population growth. The production of crops in sustainable agriculture is highly required to feed the population as it increases tremendously in this 21st century (Albahri et al., 2023). Promoting the quantity of crop production is crucial to preserve its nutritive value. Various challenges were encountered in the production of crops due to the application of chemical derivatives, which reveal the prevalence of chemical hazards in the environment (Nguyen et al., 2023). Thus, the a demand for sustainable means of promoting a friendly approach to improving the agricultural system. Integrating lesser crops, also known as underutilized crops, into potential food systems is highly significant. The process of breeding, including molecular and conventional approaches, has been utilized to improve the production of food crops, yet to meet up with food production is far fetch (Jabran et al., 2023).

Implementing genome sequences is significant for comprehending the biochemical and physiological methods of regulating plant stimulus to biotic and abiotic stresses and plant traits (Raja and Vidya, 2023). The phylogenesis of sequence technologies has led to the production of voluminous plant genome data (Xia et al., 2023). This has produced the possibility of introducing technology in crop promotion via technological advances. In this modern technology of life science, novel observation has been found in clarifying genetic functions and their pathways in line with omic technologies and bioinformatics tools employed to analyze sequence data (Li et al., 2023a). The process has been utilized in genome-wide association studies (GWAS), genomic selection, quantitative linkage locus (QTLs), population genetics, genome editing, marker-assisted breeding, and SNP genotyping detection. The mentioned processes have been modified for the category of crops. Crops, including legumes (Adeleke and Babalola, 2022) and groundnuts (Ajilogba et al., 2022a) have been suggested to assist in the food security sector in sub-Saharan Africa. These crops contain proteins and minerals that make them essential in contributing to the healthy living of people feeding on them, thereby reducing malnutrition and food insecurity. Legumes are majorly cultivated in West African countries (Nigeria, Senegal, Cote d’Ivoire, Togo), and other eastern countries like India and Indonesia (Ajilogba et al., 2022a).

The crops are well known for their potential to fix nitrogen, improving soil fertility (Kebede, 2021). They are also known to undergo crop rotation and their ability to be cultivated without the application of chemical products (Pergner and Lippert, 2023). In rural and other isolated areas, the application of chemical fertilizers is not imminent. So, these crops were relied upon for the fixation of nitrogen into the soil. The richness in nutrients, the diversity of genetic resources, and mineral composition were some of the activities of the crop to improve the soil. Legumes nodulate with the aid of nitrogen-fixing bacteria and further promote nitrogen fixation in the soil (Cheng et al., 2023).

The diversification of crops via the involvement of underutilized crops especially legumes into significant cropping systems will promote food availability (Tan et al., 2020). However, the activities taking place in soil have revealed the immense impact on the production of crops and the effect of various environmental stresses. The rhizosphere soil of these crops contains some microbes that carry out the potential to improve the growth of plants either directly or indirectly. Among these microbes are bacteria that exist as beneficial or pathogenic. The beneficial types are plant growth-promoting microbes (PGPM) that contribute to plant growth and abundant production of crops. There is a symbiotic relationship between these soil microbes, especially rhizobacteria and leguminous plants (Fasusi et al., 2023). These rhizobacteria penetrate the root nodules of the leguminous plants and convert nitrogen from its free state into nitrate that the plant can utilize by nitrogen fixation process. This process assists legumes to flourish especially where nitrogen is deficient in the soil and likewise improves the soil by enhancing the health status of other plants while practicing crop rotation (Hyder et al., 2023). The potential of bacteria species in the rhizosphere consists of the advantages to humans like the significant function they perform in biogeochemical cycles of the major elements like carbon, nitrogen, and sulfur and minor elements including iron, Nickel, and Mercury (Martínez-Martínez et al., 2023; Noor et al., 2023). The activities improve the engagement of microbes, including plant growth-promoting rhizobacteria (PGPR) and plant growth-promoting fungi (PGPF), in changeable energy embedded in the soil. Microbes manufacture phytohormones like Auxins and other substances like vitamins, enzymes, and other materials contributing to the growth of plants and inhibition of phytopathogens (Solomon et al., 2023). The communities of microbes in the rhizosphere are attributed to the features of soil, plant type, and plant growth stages.

The association of PGPR and leguminous crops has emphasized the potential of genomics. This gives tremendous insight into microbial functions and their genetic makeup. Researchers have determined the specific genes used in the process of nitrogen fixation and likewise the growth-promoting characteristics (Chieb and Gachomo, 2023; Fahde et al., 2023; Kim et al., 2023). Through the study of genomics, scientists identified and characterized major genes that partake in this mutualistic relationship of legumes and rhizobia. This study also presents the discovery of signaling molecules used in communication and recognition between rhizobia and legumes (Khan et al., 2023). A typical example is observed in the production of chemical substances called flavonoids by leguminous plant roots that attract rhizobia to the plant and nod factors, a chemical substance produced by the rhizobia activate the production of nodules in leguminous roots. In addition, genomics also unveil some PGPR which researcher analyze and make comparison of their genetic variation that contribute to plant growth-promotion potential. This knowledge does assist to optimize and choose various microbial strains that contribute to nitrogen fixation (Yao et al., 2023).Biotechnological and novel genomic processes in plants can provide solutions that can reveal the effectiveness of the objectives (Figueiredo et al., 2023). Novel techniques comprising biotechnology and the production of bio-based material can perform the function of improving agricultural sustainability. This is because they are safer for consumer use and for the environment. Biotechnology proffers resolutions for various problems of the modern world by inserting bioenergy, biopharmaceuticals, biofuels, biomaterials, foods, and its products for selling.

This review reveals the importance of plant genomics in bioeconomy promotion and the possibilities brought about by plant breeding techniques. Due to the challenges of underdevelopment and changes in the average weather conditions, the problem of food scarcity has not been resolved. So, the advancement of crop production suggests how to combat the problems. The review further reveals the advantages of the beneficial microbes in crop growth promotion and describes the application of omic techniques to characterize plant-microbe interaction.

### Legume-PGPR interaction

Plants preserve the complex environment by stimulating root exudates into the soil environment (Paponov et al., 2023). Plants produce substances that are products of photosynthesis, especially in the roots embedded in the soil. As a result, the region of soil containing carbon increases the population of microbes, unlike bulk soil which lacks carbon substances. A higher concentration of microbial communities in the soil is a result of interaction between plants and the roots that reveal various associations (Wang et al., 2023b). Some attributes measure the amount and grade of root exudates, plant growth stages (McLaughlin et al., 2023), abiotic stress (high temperature and drought) (Shaposhnikov et al., 2023), soil type (Wang et al., 2023a), phytopathogen invasion (Tortella et al., 2023), and the status of plant feeding (Heuermann et al., 2023). There will be a proposition of promoting beneficial microbes if the production of root exudates is feasible. Amino acids, sugar, phenolics, organic acids, etc., are some of the materials present in the root exudates (Agarwal et al., 2023). These carbon source substances draw the advantageous and nonadvantageous microbes into the rhizosphere soil region (Magnoli and Bever, 2023). The advantageous microbes keep plants safe against phytopathogens via direct and indirect mechanisms (Fahde et al., 2023). Yet, the carbon compounds likewise draw pathogenic microbes, which eventually lead to competition for nutrients, cause diseases in plants, and contribute to rhizobiome structuring (Adedayo and Babalola, 2023). Some plants have shown how microbial communities dwelling in their rhizosphere attract microbes that harbor protection on plants and inhibit pathogen invasions in the rhizosphere (Nwachukwu and Babalola, 2022). The physiochemical and biological features carry out significant functions in the association of plants and microorganisms. Even though phytopathogens have an immense effect on plant health, some beneficial microbes living in the rhizosphere or the tissues of plants contend with phytopathogens for nutrients and space, so end up in biocontrol activities beneficial against pathogenic organisms (Cao et al., 2023).

PGPR are found and multiply in the rhizosphere and tissues of healthy plants and improve plant growth, and abundant crop production, prevent plants against phytopathogens, and inhibit or eradicate biotic and abiotic stresses (Ojuederie et al., 2019; Olanrewaju et al., 2019). A direct form of microbial interaction with plant host is obtained in growth promotion, while the indirect form involves biological control potential against phytopathogens. Direct mechanisms involve phytohormone stimulation that reveals inhibition or promotion of the growth of plant roots, induces systemic resistance, promotes the acquisition of nutrients, and prevents crops from biotic and abiotic stress (Olanrewaju et al., 2017). Due to these properties, PGPR was accepted by researchers as an alternative to chemical fertilizers and other derivatives. Thus, PGPR, including *Bacillus thuringiensis*, contributes to abundant crop production and improves the health of plants, as reported by (Adeniji et al., 2021). Developing plants decrease the availability of Iron (Fe) by assimilating Fe and produce exudates that lure beneficial microbes into the soil to assimilate the Fe (Hyder et al., 2023). Certain PGPR is responsible for the production of siderophores in Fe-stressed environments and is enriched to inhibit phytopathogens via competition for Fe (Sarker et al., 2021). Plants utilize iron-bound siderophores that contribute to their growth (Swarnalatha et al., 2022). This reveals that the microorganisms producing Siderophore can avail the Fe needed by plants because Fe is a component of chlorophyll that is highly required for the photosynthesis process (Molnár et al., 2023). Another form applied to the reduction of plant diseases is the capability of microbes inhabiting the rhizosphere to inhibit soil or compost from disallowing the invasion of phytopathogen.

### Symbiontic association of leguminous plant and rhizosphere microbes

**FIGURE 1:**
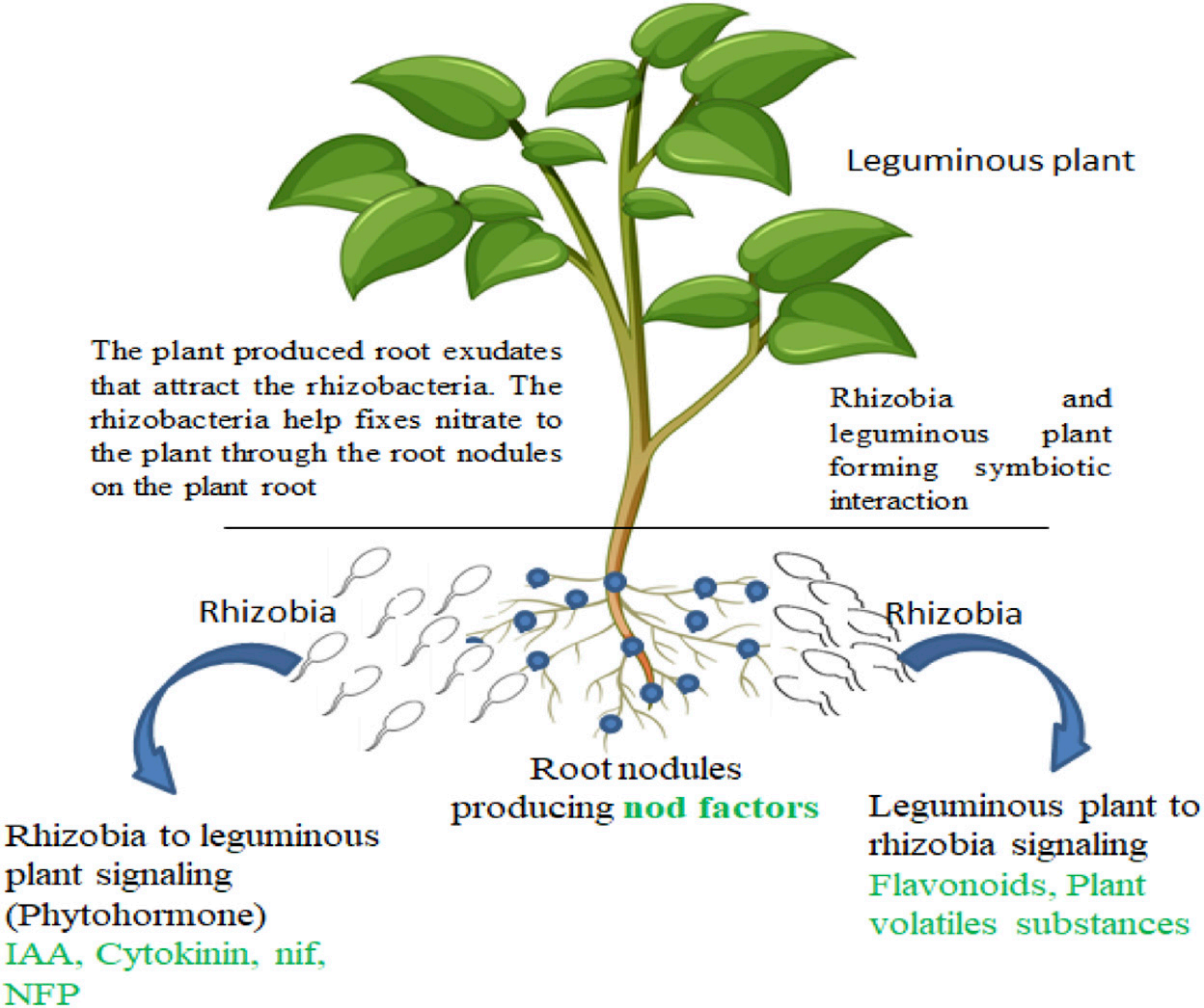
Interaction of rhizobia with leguminous plants.

Rhizosphere microbes and leguminous plants have beneficial relationships (Figure 1). Their interaction inhibits or eradicates the invasion of the phytopathogens on the plants. Rhizobia species, including *Azorhizobium*, *Allorhizobium*, *Bradyrhizobium*, *Mesorhizobium*, and *Sinorhizobium*, are common examples inhabiting the rhizosphere of leguminous plants (Babalola et al., 2017). The symbiotic association leads to the acquisition of nitrogen in the soil. Leguminous plants produce nodules and fix nitrogen acquired in the soil during the symbiotic association with *Bradyrhizobium* strains. The production of a nodule in legume plants while interacting with rhizosphere microbes leads to the production of some compounds like flavonoids, betaines, and aldonic acid in the form of root exudates. The exudates signal the PGPR in a mutual association that otherwise promotes the production of the nod gene that stimulates nodulation by associating with the nod proteins present in the PGPR cell wall (Wang et al., 2018; Walker et al., 2020). In the root hair of leguminous plants, the physical effects were revealed when the PGPR (rhizobia) responded to the production of oligosaccharide nod factors. This results in the production of nodules that eventually promote nitrogen fixation. PGPR, especially rhizobia, contributes to plant growth and improves the development of seed by producing nod factors.

### Non-symbiotic association of leguminous plants and rhizosphere microbes

Another form of association undergone by leguminous plants is the stimulation of phytohormones by the PGPR and the promotion of plant growth and abundant production of crops. Besides, leguminous plants do produce phenolics that aid the inhibition of phytopathogens, provision of nutrients to plants, and beneficial microbial growth (Egamberdieva et al., 2023). Phenolic compounds produced arbitrate the nod gene elicitors production. Nod gene elicitors control the stimulation of nod factors on the plant roots (Ahmed et al., 2020). After being collected in the rhizosphere, it results in the biological synthesis of flavonoids, which leads to the production of a higher concentration of phytoalexins for plant protection against spoilage organisms, and more reported in Table 1. Riboflavin (Vitamin B2) can likewise be produced by rhizobia and change photochemically (Lopez et al., 2019). Rhizobia has been reported to inhibit the growth of bacterial and fungal spoilage organisms affecting other crops, including soybean (Paravar et al., 2023), okra (Sharma, 2019), tomato (Adedayo et al., 2023a; Adedayo et al., 2023b), sunflower (Adeleke et al., 2021), Bambara groundnut (Ajilogba et al., 2022c), etc. Root exudates, including organic acid anions and phytosiderophores, are significant in the availability of minerals and their presence in agricultural soil (Wang et al., 2023c).

**TABLE 1:** Microbial beneficial effects on leguminous crops.

Leguminous crops	Microbes	Activities	References
*Vigna subterranean*	*Pseudomonas* spp.	Contributes to plant growth and prevents disease invasion	Ajilogba et al. (2022a)
*Vigna subterranean* and *Arabidopsis thaliana*	*Rhizobium*, *Pseudomonas*, *Azospirillum* and *Bacillus*	Promote the abundant production of legumes and other crops by fixing nitrogen into the soil	Igiehon and Babalola (2018)
*Phaseolus vulgaris*	*Kosakonia cowanii*, *Pantoea agglomerans*, *Variovorax paradoxus*, *Staphylococcus aureus*, *Enterobacter ludwigii*, and *Bacillus pumilus*	The endophytic microbes help fix plant nutrients, thereby enhancing the growth of plants	Ayilara et al. (2023)
*Vigna unguiculata* and *Arachis hypogaea*	*Bacillus* spp., *Pseudomonas* spp., *Streptomyces* spp.	The soil microbes are involved in nutrient mineralization, and so promote plant growth	Aremu et al. (2017)
*Vigna unguiculata, Phaseolus vulgaris, Arachis hypogaea*	PGPR	Aid biological nitrogen fixation that enhances the growth of leguminous crops	Babalola et al. (2017)
	*Bacillus* spp., *Pseudomonas* spp.		
*Vigna subterranean*	*Bradyrhizobium japonicum*	The PGPR contributes to the abundant production of groundnut compared to N-fertilizer	Bitire et al. (2022)
*Vigna subterranea*	*Stenotrophomonas maltophilia*, *Chryseobacterium* spp., *Pseudomonas alcaligenes*, *Pseudomonas plecoglossicida*, and *Pseudomonas hibiscicola*	These microbes have the capacity to produce indole acetic acid (IAA) and the potential to solubilize phosphate	Ayangbenro et al. (2023)
*Vigna subterranea*	*Rubrobacter*, *Acidobacterium*, and *Skermanella*	The diversity and functions of the microbes attributed to the growth of the crop	Ajilogba et al. (2022b)
*Vigna subterranean*	*Rhizobia*	The microbes promote the potential of the crops to resist abiotic conditions, prevent disease invasion, and ensure the fixation of nitrogen	Ayilara et al. (2022)
*Vigna subterranean*	*Bacillus*, *Pseudomonas*, and *Streptomyces*	The microbes improve crop growth and prevent the invasion of diseases on the plant	Olanrewaju and Babalola (2022)
*Vigna subterranean*	*Bradyrhizobium japonicum*	The PGPR promotes nitrogen fixation and further improves plant growth	Bitire et al. (2023)
*Glycine max*	*Rhizobium cellulosilyticum* and *Arbuscular mycorrhizal*	The microbes contribute to the growth of the plant in the semi-desert region	Igiehon et al. (2021)
*Vigna subterranea*	*B. amyloliquefaciens*, *B. thuringiensis* and *Bacillus* sp.	The PGPR contribute to the production of volatile organic compound that acts as antibacterial against phytopathogens	Ajilogba and Babalola (2019)
*Vigna subterranean, Glycine max, Vigna unguiculata, Phaseolus vulgaris, Arachis hypogaea*	PGPR and PGPR traits	The rhizobacteria enhance plant health by promoting plant growth, preventing the occurrence of phytopathogens through biological control activities, and improving soil health	Hassen et al. (2023)

### Molecular mechanisms applied in the study of PGPR

Certain biochemical and cultured-based tests have been employed for a long time to study PGPR (Utami et al., 2022). With recent progression in molecular technology, various gene technological data, including genomics, metagenomics, transcriptomics, and proteomics data, are uploaded on global websites (Lata et al., 2023). Genomic sequence analysis of PGPR is grouped into two groups, and they are whole genome sequencing (WGS) and specific genome sequencing (SGS) (Simlat et al., 2023).

WGS reveals all the chromosomes and plasmids present in the PGPR. WGS-employed next-generation sequencing (NGS) shows the appropriate genetic makeup of organisms (Kurotani et al., 2023). Whereas SGS revealed only a certain part of the genome employed for comparison and characterization (Lim et al., 2023). The particular gene in this group is 16S rRNA used to identify bacteria origin to specific levels of organization (Abramova et al., 2023). Another form of identifying the bacteria is an intra-genomic spacer (IGS) 16S-23S, repetitive sequence-based PCR (rep-PCR) that amplify and sequence highly conserved repeats. Restriction fragment length polymorphism (RLFP) analysis has been used to categorize PGPR (Li et al., 2023b).

Some genes, including *atpD*, *dnaK*, *dnaJ*, *gap*, *glnll*, *gltA*, *gyrG*, *pnp*, *recA*, *rpoA*, and *thrC* were employed in strain typing involved in multilocus sequence analysis (Swarnalakshmi et al., 2020). Absolute primers are employed to intensify genomic sequences randomly, known as random amplified polymorphic DNA sequencing. To amplify particular genes together with a secondary mode of sequencing using restriction profiling and denaturing gradient gel electrophoresis (DGGE) to reduce the assembly of mismatches in limited regions or variation in genome GC content (Nakatsu, 2021). The process of fixing nitrogen in the soil by the biological process is a significant method of promoting PGPR with the aid of nitrogenase enzymes producing genes like *nifD*, *nifK*, and *nifH* genes (Özdoğan et al., 2022).

Moreover, PGPR partakes in the solubilization of phosphorus in the production of gluconic acid, which makes use of glucose dehydrogenase. Apart from the mentioned characteristics, PGPR stimulates phytohormones like gibberellin, ethylene, indole acetic acid (IAA), abscisic acid, and cytokinin (Ojuederie et al., 2019; Koza et al., 2022). However, certain genes like *ipdC* and *amlE* are responsible for IAA production, as found in other phytohormones (Adedayo et al., 2023a). For iron utilization, siderophores were produced by PGPR which is regulated by the Siderophore gene. *Pseudomonas* spp. possess membrane receptor that codes for the *pupA* gene, which transports the Siderophore complex in the cell (Sah et al., 2021).

### Plant growth-promoting genes contributing to the growth of leguminous plant

Leguminous plants have a special interaction with PGPR especially rhizobia as discussed above. This interaction yields the fixation of atmospheric nitrogen to a plant-utilizable form that contributes to the healthy status of the plant, which is important for plants that have no access to nitrate assimilation (Figure 1). The growth-promoting genes are mainly associated with the production and upkeep of mutualistic association between rhizobia and leguminous plants and the following genes discussed below are responsible for this process.

Nodulation (nod) genes: are the genes used for starting and regulating the production of nodules which is the complex body where the organism (rhizobia) dwells (Malik et al., 2020). The genes examples are *nodA, nodB, nodC,* and *nodD*.

Nitrogen fixation (nif) genes: they are involved in the production and roles of nitrogen-fixing nodules. They also carry out potential action in synthesizing nitrogenase enzyme that converts natural nitrogen gas to ammonia and their example are *nifD, nifH*, and *nifK* (Adedayo et al., 2023a)

Auxin biosynthetic genes: are genes responsible for the production of hormones that contribute to the growth of plants. The stimulation of these hormones is promoted by some rhizobia thereby contributing to the elongation of plant roots and production of the root nodules. The typical examples include indole-3-acetamide hydrolase (*iaah*), an enzyme responsible for the production of auxin, and indole acetic acid (*IAA*) which is a hormone (Adedayo et al., 2023a).

Cytokinin biosynthesis genes: these are responsible for the production of plant hormone Cytokinin. The hormone contributed to the division of cells as well as their differentiation (Šmeringai et al., 2023). Various rhizobia are producing this hormone that contributes to the production of nodules. These genes also contributed to the biological synthesis of cytokines with the aid of the isopentenyl transferase (ipt) enzyme for plant growth.

Plant receptor genes: these are genes constituting particular receptors to identify and associate with the signals produced by rhizobia. Examples include nod factor rhizobia did produces LysM Receptor Kinase (*NFR*) and Nod Factor Perception (*NFP*) genes (Geurts and Huisman, 2023). The genes explained above contribute to structural signaling pathways and their association to produce advantageous and mutualistic associations between leguminous plants and PGPR (rhizobia). They also enhance the development of the plants by supplying them with fixed nitrogen and assimilation of nutrients in the rhizosphere soil.

### Plant breeding

The integration of plant and animal production systems to promote environmental quality and natural resources to improve economic viability is defined as sustainable agriculture (Fatima et al., 2023; Liu, 2023). It is also defined as the application of technologies and practices that do not harm the food quality, environment, and production (Gamage et al., 2023). The significant issue of minimizing the application of non-renewable material, e.g., phosphates, crop genotypes, and promoting sustainability of agriculture (Jha et al., 2023). The utilization of modern methods of genetic breeding, manipulation and redesign, and agricultural and ecological management incorporating crop selection is sustainable development (Hemalatha et al., 2023). Agricultural practices nowadays are based on different forms that contain input-intensive systems that make use of energy, fertilizer, oil, water, etc., unlike low-input systems, which bring about low production of crops.

So, scientists’ stance should focus on plant breeding which will cut down the rate at which energy is utilized for plant production yet ascertain the production of high-quality food products. Due to climate change and the growth of the population around the globe, plant breeding requires water and, likewise, reduces the rate of water loss in other to promote the potential of sustaining water deprivation and avail food for developing populations. To promote the effectiveness of water use and production of abundant yield, the following features are required, and they include; physiological and molecular modification, osmoregulation, improved plant root system, and improved stomatal conductivity (Woźniak-Gientka and Tyczewska, 2023). Energy in the form of oil and gas is important for modern-day high-input agricultural systems. However, the energy implored for the production of crops during the period of harvest and post-harvest incorporates the application of farm machinery for cultivation, preparation of soil, harvesting, transport of the yield, and post-harvest storage. Although, it has been noted that 15% of utilized energy is demanded by the application of chemical derivatives on farmland and transportation, according to Attfield et al. (2023). The application of chemical fertilizers is regarded as not the best means when studying sustainable agriculture. It is significant to note the breeding process to improve the resistivity and tolerance in agricultural practices to boost sustainability, where the application of chemical pesticides is reduced (Esuola et al., 2016).

Various scientific processes that aid plant feature identification include genetic engineering, hybridization, tissue culture, and plant breeding (Woźniak-Gientka and Tyczewska, 2023). The benefit of genome editing (CRISPR-Cas9) is to supply high target specificity with high precision and speed (Sahel et al., 2023). Furthermore, applying this technique does not yield transgenic plants due to its inability to transfer genes to the host plants. Applying this technique, some features, including biotic and abiotic features, were tolerated to obtain new varieties of crops to improve the abundance of agricultural products (Anguita-Maeso et al., 2023). The process involved in agrobiotechnology is employed to produce Biofertilizers, biopesticides, bioherbicides, etc., in the agricultural field (Woźniak-Gientka and Tyczewska, 2023). These compounds aid in the regulation of soil toxicity and are safe for environmental use. The growth and development of plants mainly depend on the availability of macroelements, including nitrogen (N), phosphorus (P), and potassium (K) (Bitire et al., 2023), and microelements like molybdenum (Mo), iron (Fe), zinc (Zn), manganese (Mn), copper (Cu), boron (B), etc. (Banerjee et al., 2023).

### Plant breeding techniques improve the production of crops

The challenge of making available food crops to feed the developing population is the economic, social, and environmental systems of farming that oppose the adaptation of climate, degradation of the agricultural field, and loss invented on biodiversity of the microbial communities. The production of food to support the population is not sufficient as the population increases. Due to this challenge, some procedures were adopted in new breeding techniques to enhance the production of crops through genetically modified organisms (GMOs) (Rahman et al., 2023). However, the usual form of biotechnology in agricultural practices is the cultivation of new crops, also called genetically modified or engineered crops. Some of the genome-edited crops do not shelter foreign DNA (Olanrewaju et al., 2021). CRISPR-Cas9 is a genome-editing tool employed in plant study and its growth globally to improve the features of crop quality, activities against chemical derivatives like pesticides and herbicides, and disease resistance (Marone et al., 2023). Employing a CRISPR technology breeder could promote tolerance to abiotic stresses like extreme temperature and drought and improve nutrient content. Legumes have been monitored upon the application of this technology (Puppala et al., 2023). Climate change has caused extreme heat and drought that affect the production of crops to feed the global populace (Omotoso et al., 2023). CRISPR/Cas9 tools have been employed in crop plantation that requires water for their growth and cognition of significant genes to control drought stress (Banerjee et al., 2023).

### Transcriptomics analysis of PGPR dwelling in the rhizosphere of leguminous plants

This involves the genetic expression study pattern of the rhizobia to obtain knowledge about their interaction and their roles in the rhizosphere soil. PGPR are known to be involved in the significant function of improving the growth of plants and likewise promoting the availability of the nutrients required via nitrogen fixation and solubilization of phosphate (Adedayo et al., 2022).

To carry out the analysis of transcriptomes on samples, scientists isolate the specific bacteria species in the soil sample of the leguminous plants. The microbes were lysed to extract the RNA from the bacterial cell and further purified (Saleh-Lakha et al., 2005). The RNA is a precursor for transcribed genes for a specific period and as a starting medium for downstream analysis. Then, the mRNA is converted to complementary strand DNA (cDNA) with the help of an enzyme called reverse transcriptase (Ying, 2003). The cDNA stands for the expressed gene of the microbial isolate.

High throughput sequencing technologies like RNA Seq can be used to collect sequence data of cDNA molecules and further subjected to analyzing the data using bioinformatics tools (Shiau et al., 2023). This constitutes aligned sequences to the reference database or genome of the recognized genes to confirm the genes expressed (Tables 2, 3). Also, the analysis of differential gene expression can be investigated to analyze various conditions of the stages of gene expression, like different rhizobia strains, rhizosphere soil they inhabit, and time range. The differentially identified genes can make available the knowledge of molecular procedures of rhizobacteria’s mutualistic association with the leguminous plants (Li et al., 2023c). These genes can contribute to the following mechanisms including; plant growth promotion, nitrogen fixation, stress tolerance, and nutrient cycling, among other functional characteristics of interest. This analysis can likewise be joined together with other high throughput omics technologies like metabolomics and proteomics to get more knowledge of PGPR community structure, functions, and potential influence on abundant production of legumes and improvement of leguminous plant health. On a short note, transcriptomics technology can assist scientists in decrypting the genetic makeup of plant-microbe interactions and make available cogent information for sustainable agriculture. The characteristics of *Paenibacillus polymyxa* (Table 2) and *P. agglomerans* (Table 3) were revealed showing various characteristics of the PGPR ranging from growth features to genomic size of various strains strain isolated. *Pantoea agglomerans* displayed various potential including plant growth promotion, phosphate solubilizing potential, secretion of hormones like IAA and Siderophore, and synthesis of acetoin and 2,3-butanediol according to Shariati et al. (2017).

**TABLE 2:** Plant growth-promoting potential of *Paenibacillus polymyxa* contributing to the growth of crop plants like tomatoes (Xie et al., 2016).

Growth parameter	Control^a^	1–18	1–49	1–43	TD94	WLY78
Shoot length (cm)	22.76 (±1.74)	28.16 (±1.13)**	27.57 (±2.37)**	26.34 (±0.49)**	26.13 (±0.62)**	26.43 (±1.07)**
Root length (cm)	12.93 (±1.93)	13.17 (±1.59)	13.85 (±1.18)	13.02 (±0.75)	13.63 (±0.77)	13.03 (±0.75)
Fresh weight (g)	2.68 (±0.71)	4.05 (±0.43)**	3.96 (±0.62)	3.68 (±0.45)**	3.57 (±0.26)**	3.50 (±0.32)**
Dry weight (g)	0.19 (±0.05)	0.31 (±0.03)**	0.30 (±0.05)**	0.29 (±0.02)**	0.29 (±0.02)**	0.27 (±0.02)**

^a^ Signifies tomato plant.

**Reveals the level of significance (*p* > 0.01).

**TABLE 3:** Comparative genomic analysis of *Pantoea agglomerans* strain as plant growth-promoting rhizobacteria according to Shariati et al. (2017).

Strains	Genome size (Mb)	GC (%)	No. of rRNA	No. of tTRNA	Scaffolds	No. of genes	No. of proteins	GenBank Accession no.
P5	5.16726	55.4	7	63	150	4,745	4,674	GCA_002157425.2
Tx10	4.85699	55.1	48	142	22	4,627	4,500	GCA_000475055.1
Eh318	5.03584	54.8	28	73	34	4,791	4,627	GCA_000687245.1
190	5.00257	55.1	24	77	5	4,878	4,778	GCA_000731125.1
MP2	4.73383	55.2	22	71	16	4,352	4,214	GCA_000757415.1
IG1	4.83958	55.0	2	63	18	4,443	4,341	GCA_000241285.2
299 R	4.58148	54.3	27	63	109	4,267	4,157	GCA_000330765.1
RIT273	5.36534	55.1	17	76	26	4,999	4,914	GCA_000627115.1
DAPP-PG734	5.36593	54.7	36	70	195	5,107	5,107	GCA_000710215.1
4	4.8102	55.1	19	72	12	4,370	4,260	GCA_000743785.1
LMAE-2	4.98116	55.1	51	79	155	4,681	4,406	GCA_000814075.1

## Conclusion

The sustainability of agricultural soil concerns the agricultural development and application of fertilizers to improve farming activities. Major nutrients required to boost the fertility of the soil and encourage plant growth, including nitrogen, phosphorus, and potassium, were discussed in the context. Nevertheless, PGPR contributes to the health status of plants, thereby contributing to the healthy growth of plants and improving the abundant production of crops and generally regarded as safe for humans, plants, and the environment. Due to these features, they possess, they are alternatives to chemical fertilizers. The diversity of microorganisms in soil contributes to plant growth and improves their various ecosystem functions in the soil. The plant root produces some substances called root exudate that attract soil microbes and reveal the microbial diversity inhabiting the soil. So, high throughput sequencing involving genomics is used to identify unculturable microbes. However, some factors, including soil types and plant genotypes, control the activities of PGPR in the soil. So, genetic variation of advantageous PGPR interaction with plants can be incorporated into plant breeding to boost the production of legumes for agricultural sustainability.
